# Supplementary material for: The interactome of intact mitochondria by cross-linking mass spectrometry provides evidence for coexisting respiratory supercomplexes
Source: Mol Cell Proteomics. 2017 Dec 8;17(2):216–32. doi: 10.1074/mcp.RA117.000470 (PMC5795388; doi:10.1074/mcp.RA117.000470)
Supplement: Supplemental Data [file supp_RA117.000470_133922_0_supp_23978_fzffwf.zip › spectra_annotation/mito_spectra_annotation/337-1-5-1-5-1.pdf]

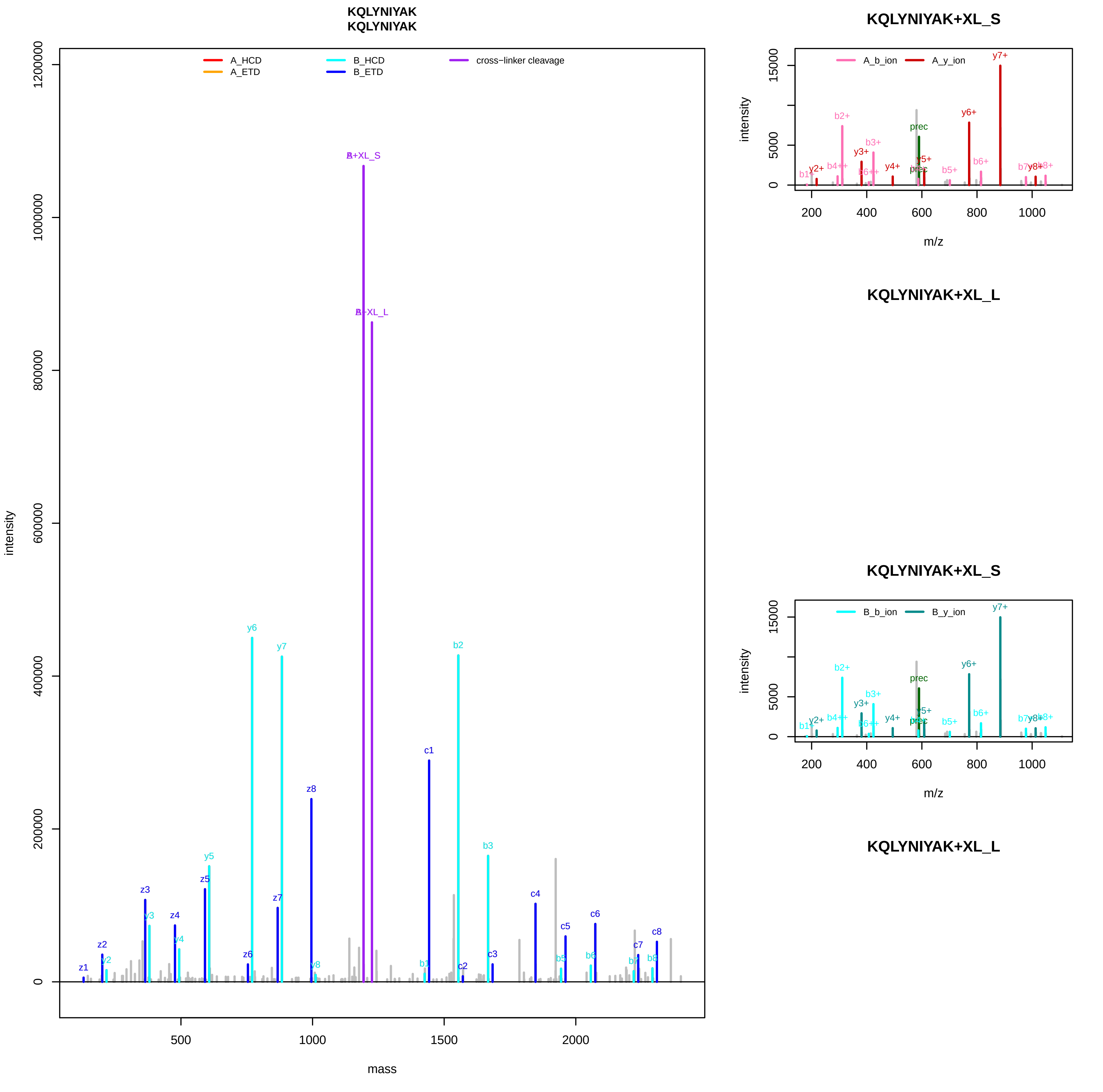

KQLYNIYAK
KQLYNIYAK
A_HCD
A_ETD
B_HCD
B_ETD
cross−linker cleavage
intensity
mass
0
200000
400000
600000
800000
1000000
1200000
500
1000
1500
2000
z1 z2 y2 z3 y3 z4 y4 z5 y5 z6 y6 z7 y7 z8 y8 c1 b1 c2 b2 b3 c3 c4 b5 c5 b6 c6 b7 c7 b8 c8
KQLYNIYAK+XL_S
A_b_ion
A_y_ion
b1+ y2+ b4++ b2+ y3+ b3+ b6++ y4+ prec y5+ b5+ y6+ b6+ y7+ b7+ y8+ b8+
m/z
KQLYNIYAK+XL_L
KQLYNIYAK+XL_S
B_b_ion
B_y_ion
b1+ y2+ b4++ b2+ y3+ b3+ b6++ y4+ prec y5+ b5+ y6+ b6+ y7+ b7+ y8+ b8+
m/z
KQLYNIYAK+XL_L
